# A pilot randomized controlled trial of a tailored cognitive behavioural therapy based intervention for depressive symptoms in those newly diagnosed with multiple sclerosis

**DOI:** 10.1186/s12888-016-1152-7

**Published:** 2016-12-07

**Authors:** Litza A. Kiropoulos, Trevor Kilpatrick, Alex Holmes, Jennifer Threader

**Affiliations:** 1 Melbourne School of Psychological Sciences, University of Melbourne, Victoria, 3010 Australia; 2 Psychology Department, Royal Melbourne Hospital, Parkville, Victoria Australia; 3 Melbourne Brain Centre and MS unit, Royal Melbourne Hospital, Parkville, Victoria Australia; 4 Centre for Neuroscience and the Melbourne Neuroscience Institute, University of Melbourne, Parkville, Victoria Australia; 5 Florey Neuroscience Institute of Neuroscience and Mental Health, University of Melbourne, Parkville, Victoria Australia; 6 Department of Psychiatry, University of Melbourne, Parkville, Victoria Australia

**Keywords:** Depression, Cognitive behavioral therapy (CBT), Multiple sclerosis, Newly diagnosed, Early intervention, Anxiety

## Abstract

**Background:**

To examine the effectiveness and acceptability of an 8-week individual tailored cognitive behavioural therapy (CBT) intervention for the treatment of depressive symptoms in those newly diagnosed with multiple sclerosis.

**Methods:**

The current study presents a pilot, parallel group randomized controlled trial (RCT) with an allocation ratio of 1:1 conducted in a large research and teaching hospital in Melbourne, Australia. 30 individuals with a mean age of 36.93 years (SD = 9.63) who were newly diagnosed with multiple sclerosis (MS) (X = 24.87 months, SD = 15.61) were randomized to the CBT intervention (*n* = 15) or treatment as usual (TAU) (*n* = 15). The primary outcome was level of depressive symptoms using the Beck Depression Inventory-II (BDI-II). Secondary outcomes were level of anxiety, fatigue and pain impact, sleep quality, coping, acceptance of MS illness, MS related quality of life, social support, and resilience. Tertiary outcomes were acceptability and adherence to the intervention.

**Results:**

Large between group treatment effects were found for level of depressive symptoms at post and at 20 weeks follow-up (*d* = 1.66–1.34). There were also small to large group treatment effects for level of anxiety, fatigue and pain impact, sleep quality, MS related quality of life, resilience, and social support at post and at 20 weeks follow-up (*d* = 0.17–1.63). There were no drop-outs and participants completed all treatment modules. All participants reported the treatment as *‘very useful*’, and most (73.4%) reported that the intervention had addressed their problems *‘completely’*.

**Conclusions:**

These data suggest that the tailored early intervention is appropriate and clinically effective for the treatment of depressive symptoms in those newly diagnosed with MS. A larger RCT comparing the CBT intervention with an active comparative treatment with longer term follow-up and cost effectiveness analyses is warranted. The pilot trial has been retrospectively registered on 28/04/2016 with the ISRCTN registry (trial ID ISRCTN10423371).

## Background

Multiple sclerosis (MS) is the most common neurological disorder in young adults. It is estimated that 2.5 million people are living with MS worldwide. Diagnosis of MS is typically between 20 and 40 years of age, three quarters of whom are female [1]. MS has a range of consequences on mental health. Depression and anxiety have been found to be common in individuals with MS and have been reported to be at clinically high levels especially in the early stages of the illness [2–4]. The lifetime prevalence of depression among individuals with MS has been found to be 50% [5] and point prevalence rates range from 15 to 26% [6]. Similarly, anxiety affects between 16 and 45% of the MS population [7, 8] and has been associated with younger age of onset, disease severity, fatigue [9] and severity of depressive symptoms [10]. Two studies suggest that up to 36% of MS patients continue to have high levels of depressive and anxiety symptoms in the first years after diagnosis [3, 11].

CBT for the treatment of depression in people diagnosed with MS have demonstrated significant reductions in depression. A review of seven CBT studies (individual (3 studies), group (3 studies) and by computer (1 study)) found a medium treatment effect of 0.5 SD [12]. One study compared individual CBT with sertraline and group therapy and found that those in the CBT and the sertraline groups showed greater improvements in their levels of depression compared to those in group therapy. In addition, those in the individual CBT group displayed improvements in mood, coping, and suicidal ideas [13]. Askey-Jones, David, Silber, Shaw and Chalder (2013) [14] also examined the effectiveness of a CBT delivered intervention and found that CBT resulted in statistically significant decreases in depression and anxiety with large effect sizes. More recently, Fischer et al., (2015) [15] conducted a RCT comparing a CBT based online intervention with a waitlist control group for the treatment of depression in a sample of 71 outpatients who were diagnosed with MS for a mean length of 8 years. The researchers found that those who completed the 9-week online CBT program reported lower BDI-II scores at post and 6 months follow up compared to those in a waitlist control group.

To date, there are no published studies of early provision of tailored CBT for the treatment of depression in the first five years of a MS diagnosis with interventions focused on those with established disease (greater than 8 years). There are good reasons for early provision of CBT for the treatment of depression in those newly diagnosed with MS. Firstly, the time around receiving a MS diagnosis has been shown to be when individuals experience significantly higher levels of depression and anxiety [2, 4, 11, 16]. Two studies suggest that up to 36% of MS patients continue to have high levels of depressive and anxiety symptoms in the first two years after a diagnosis [11] and are at greater risk of suicide [17]. Psychological factors are likely to be contributing. For example, MS is diagnosed at a time point when individuals are typically establishing careers, relationships and families. Individuals may also be adapting to the MS diagnosis and symptoms, the burden of uncertainty and dealing with the loss of physical and cognitive functioning, changes in interpersonal relationships, social and work roles and social support and a reduction in positively reinforcing activities. Secondly, if left untreated depression will worsen and contribute to further deterioration having an impact on the course of MS [18] resulting in exacerbation of MS relapses [19] and contribute to higher suicide rates [5]. Treating depression with CBT can contribute to the alteration of disease outcomes. For example, depression has been found to be related to neurological changes in the brain due to the demyelination process [20], treatment adherence to medical advice and treatments [21], immune functioning [22] and MS disease exacerbation [23]. Therefore, early provision of treatment of depression may lead to improvements in adherence with possible positive impacts on disease processes i.e., potentially reduce exacerbations and influence longer term MS progression via increased treatment adherence and reduce markers of MS inflammation and deterioration [24]. Thirdly, studies have demonstrated that early recognition and treatment of depression can improve social function, increase productivity and decrease absenteeism in the workplace [25]. At the social level, early intervention has been supported because the longer a person remains depressed the more strained the interpersonal and occupational roles may become.

An intervention at the time of first diagnosis, with the potential to modify the trajectory of psychological morbidity, has not been explored. In light of the burden of depression in the early stages of disease, the aims of the study were to assess the efficacy of a tailored 8-week individualized CBT intervention in the treatment of depressive symptoms (primary outcome) in individuals who are within five years of a MS diagnosis. Secondary aims were to examine improvements in levels of anxiety, fatigue, pain, sleep quality, quality of life, coping, MS illness acceptance and resilience (secondary outcomes) at post and 20 week follow up time points and evaluate satisfaction and adherence to the tailored intervention (tertiary outcome).

## Methods

### Study design

This study was a 2-group RCT with an allocation ratio of 1: 1: (1) Tailored CBT intervention (*n* = 15); and (2) TAU (*n* = 15). All participants completed questionnaires at pre- and post-treatment and at 20 weeks follow up.

### Participants

#### Eligibility criteria

Inclusion criteria were: 1) having a definite diagnosis of MS from a neurologist; 2) being within 5 years of receiving a diagnosis of MS; 3) scoring at least 10 on the *Beck Depression Inventory-II* (BDI-II) [26]; 4) not currently undertaking other psychological treatment for depressive or anxiety symptoms for the length of participation in the current trial; 5) speak English fluently; 6) no current or lifetime diagnosis of psychosis; 7) no current substance dependency; 8) no gross cognitive impairment; and 9) no changes to medications prior and during involvement in the trial.

#### Recruitment

**Fig. 1:**
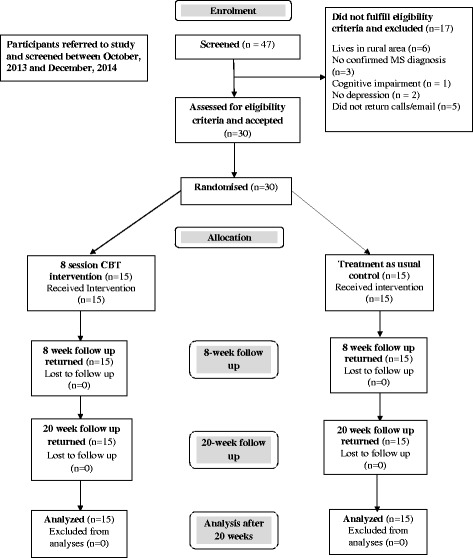
CONSORT flow chart of participants going through the trial

The study took place at a large teaching and research hospital in Melbourne, Australia. Participants were recruited through neurologists working in a large MS outpatient clinic at the hospital, advertisements on MS related websites and local press releases. All participants responded positively to two screening questions for depressive symptoms. Participants were recruited from October 2013 to December 2014. The study was approved by the Melbourne Health ethics committee. Figure 1 outlines the flow of participants through the trial and reasons for ineligibility. Forty-seven people were approached and screened and took part in the study. 17 of the 47 screened were not eligible for the trial giving a participation rate of 64%. Fifteen participants were randomly allocated to the CBT group and 15 to the TAU control group. There were no drop outs. All participants completed the pre, post and 20 week follow up questionnaires. Follow up assessments ended April, 2015. The trial was stopped after achieving a total number of 30 participants (15 in each group).

#### Randomization and allocation concealment

Participants were randomly assigned using a computer generated random numbers table using an allocation ratio of 1: 1 to 1 of the 2 treatment groups. The allocation sequence was concealed from the clinician enrolling and assessing participants in opaque, sealed envelopes that were numbered in advance and opened sequentially. All clinicians were blind to the group allocation sequence. The assignment to the CBT intervention or TAU group schedule was locked away in a locked filing cabinet at the hospital. Blinding of staff and participants after assignment to interventions was not maintained. Clinicians who delivered the intervention were also involved in providing the assessment questionnaires to participants.

#### Sample size calculation

A sample size of 30 was selected as this number has been recommended for pilot studies [27].

### Procedure

#### Pre-treatment assessment

Consenting and eligible participants were invited to undertake a clinical interview at baseline with a clinical psychologist at the hospital. One female senior clinical psychologist and one male provisional clinical psychologist undertook assessments. During this baseline interview, the goals and procedures were explained in detail and written consent was obtained from all participants. The *Structured Clinical Interview of Disorders- Research version* (SCID-I/P) [28] was used to screen out patients with psychotic disorders and substance dependency. Participants who were eligible were asked to complete a battery of questionnaires.

#### Tailored CBT intervention

The aim of the 8-week tailored intervention is to significantly decrease level of depressive symptoms (primary outcome), anxiety, fatigue and pain impact and to contribute to improvements in levels of quality of life, sleep difficulties, MS illness acceptance, active coping skills, social support and resilience (secondary outcomes) in those newly diagnosed with MS. Additionally, the pilot trial aimed to assess the adherence and acceptability among participants (tertiary outcomes) of the tailored CBT program for individuals with MS. The tailored CBT intervention lends from Beck’s cognitive theoretical model for the treatment of depression in adults. Participants were given homework to complete for each of the 8 sessions. All sessions (apart from the first session which was 1.5 h) were 1 h in duration and took place in a clinic room at the hospital. A senior clinical psychologist and a provisional clinical psychologist (supervised by the clinical psychologist) provided the intervention.

The CBT intervention consisted of 8 individual modules which focused on CBT based principles and strategies. These strategies included progressive muscle relaxation, controlled breathing exercises, pleasant activity scheduling, problem solving skills, cognitive exercises which helped individuals identify, challenge and manage unhelpful thoughts and beliefs. For the intervention, a therapy manual was developed by the first author, which included tailored modules and skills for MS patients. Relevant and targeted treatments have been found to be more effective and preferred over generic CBT interventions among individuals with MS [18]. MS specific issues such as addressing grief over MS-related losses and use of relevant examples during therapy were included in the tailored program. The intervention also focused on facilitating acceptance of MS illness and adjusting to living with MS and the final therapy session was dedicated to relapse prevention.

#### Treatment as usual (TAU)

Participants in the TAU group did not obtain any psychological treatment for depressive symptoms or anxiety for the entire length of their involvement in the trial (20 weeks) and received usual medical care from their neurologist which may include 1–3 visits for medication review during the course of involvement in the current trial. All participants were offered the tailored CBT intervention after completion of their involvement in the TAU arm and 2 participants took up this offer.

### Outcome measures

#### Demographic and medical data

Demographic data (age, gender, level of education, employment status, marital status, ethnic background); MS data (MS type, months since first MS symptoms, current disease modifying medication); mental health data (antidepressant medication use, previous diagnosis of depression and anxiety disorder); and level of ambulation was measured using the ambulation questions from the self-report Expanded Disability Status Scale (EDSS) [29] were collected from each participant at baseline.

### Primary outcome measure

#### Level of depression

The primary outcome was level of depressive symptoms at post and 3 month follow up which was measured with the *Beck Depression Inventory-II* (BDI-II) [26]. Cronbach’s α in this sample was excellent (.90).

### Secondary outcome measures

#### Level of anxiety

Anxiety was measured with the 20-item *State Trait Anxiety Inventory* (STAI) [30]. The STAI evaluates feelings of tension, nervousness, worry and apprehension ‘*in the past two weeks, including today*’ with higher scores reflecting higher severity. In the current sample, the STAI showed excellent reliability (*a* = .95).

#### Fatigue impact

*The 5-item Modified Fatigue Impact Scale* (MFIS-5) [31] was used to measure fatigue impact. Higher scores indicate greater impact of fatigue on physical, cognitive and psychosocial functioning. Cronbach’s *α* in this sample was excellent (.84).

#### Pain impact

The *Pain Effects Scale* (PES) [31] was used to measure the level of impact that pain had on mood and behavior. Higher scores indicate a greater impact of pain on a patient’s mood and behavior. Cronbach’s *α* in this sample was (.91).

#### MS related quality of life

The *Multiple Sclerosis Quality of Life* (MSQOL-54) [32] was used to measure MS related quality of life. Two summary scores, physical health and mental health, can be derived from a weighted combination of scale scores. Higher scores indicate higher quality of life. Cronbach *α* for the scale was .95.

#### Sleep quality

The 9-item *Pittsburgh Sleep Quality Index* (PSQI) [33] was used to examine sleep quality, sleep latency, sleep duration, habitual sleep efficiency, sleep disturbances, use of sleep medication, and daytime dysfunction over the last month. An overall score of 5 or more indicates a ‘poor’ sleeper. Cronbach’s *α* for this scale was .74.

#### Coping

The 66-item *Ways of Coping Questionnaire* (WCQ) [34] was used to examine coping which consists of eight scales measuring confrontive coping, distancing, self-controlling, seeking social support, accepting responsibility, escape-avoidance, planful problem-solving, and positive reappraisal. Higher scores indicating greater use of the coping strategy. Cronbach’s *α* for the entire scale was .94.

#### Acceptance of MS illness

The 10-item *Acceptance of Chronic Health Conditions Scale* (ACHC) [35] was used to measure acceptance of MS illness which has been adapted to be used with an MS population. Lower scores reflect higher levels of acceptance. Cronbach’s *α* in this sample was .86.

#### Level of social support

The 12-item *Perceived Social Support Scale* (PSSS) [36] assesses perceived social support from family, friends, and others. Higher scores indicated higher perceived social support. Cronbach’s *α* in this sample was .94.

#### Level of resilience

The 33-item *Resilience Scale for Adults* (RSA) [37] was used to measure level of resilience and investigates five main protective factors: personal competence, social competence, personal structure, family cohesion and social resources. Higher scores indicate higher levels of resilience. Cronbach’s α in this sample was .93.

#### Level of therapeutic alliance

Therapeutic alliance was measured using the 19-item *Helping Alliance Questionnaire-Version 2* (patient version) (HAQ-II) [38]. The HAQ-II total score ranges from 19 to 114, with higher scores indicating stronger patient-therapist therapeutic alliance. The cut-off point to indicate good therapeutic alliance is a score of 86 or higher [38]. Cronbach’s *α* for the scale was .93.

#### Acceptance of the CBT based intervention

A 5-item questionnaire was developed by the first author to assess how useful and acceptable the tailored CBT intervention was using the following questions: *‘Approximately what proportion of the handouts have you read?’ and ‘How useful has this treatment course been for you?’*, ‘*What were the best and worst aspects of this treatment*’ and ‘*How could this treatment be improved*?’

#### Patient satisfaction questionnaire

An 18-item questionnaire was designed by the first author to assess participants’ satisfaction with the CBT intervention. The questionnaire asked about: the number of sessions completed and whether these met their needs and expectations, the timing and content of sessions, perceived benefits of the skills taught in the intervention including the management of depressive and anxiety symptoms, identifying unhealthy thinking styles, pleasant activity scheduling, mood and anxiety monitoring, challenging negative thoughts, controlled breathing and relaxation exercises, fatigue management, sleep hygiene, problem solving skills and relapse prevention. Participants were also asked whether they would recommend this intervention to a close friend in the same situation.

### Statistical analyses

Statistical analyses were performed using SPSS version 22 for Windows. To examine group differences at baseline individual analyses of variance (ANOVAs) were performed with all outcome variables. To examine group differences at post and 20 week follow up assessment, individual analyses of covariance (ANCOVAs) were performed with baseline treatment outcome scores used as the covariate. Effect sizes from pre to post and pre to follow up were calculated using Cohen’s *d* for each outcome measure. All statistical analyses included a sample of 30 participants at pre, post and 20 weeks follow up and were undertaken controlling for gender and ambulation status.

## Results

### Participants

Table 1 shows the characteristics of participants. The groups were well matched on all demographic and medical variables. All participants had relapse remitting MS and were able to walk independently without an aid. Participants were most commonly female, employed, tertiary educated, in a stable relationship, and receiving disease modifying medication. Half of the participants have been previously diagnosed with depression and almost half have previously been diagnosed with an anxiety disorder.

**Table 1:** Baseline demographic characteristics of MS participants in the CBT and TAU groups

	CBT intervention (*n* = 15)	TAU (*n* = 15)
Age (M, SD)	34.60 (9.06)	39.27 (9.93)
Time since diagnosis in months (M, SD)	26.20 (15.58)	23.53 (16.06)
Months since first MS symptoms (M, SD)	35.54 (16.47)	30.57 (18.68)
Gender (n)% female	(13) 86.7%	(9) 60%
Years of education (n)%		
Secondary	(4) 26.7%	(3) 20%
Trade, Tafe or Diploma	(4) 26.7%	(4) 26.7%
Undergraduate	(3) 20%	(2) 13.3%
Postgraduate	(4) 26.7%	(6) 40%
Employment status (n)%		
Unemployed	(4) 27%	(5) 33.3%
Part time	(4) 27%	(4) 27%
Full time	(7) 47%	(6) 40%
Marital status (n)%		
Single	(2) 13%	(0) 0%
Partnered/Married	(13) 86.6%	(15) 100%
Ethnic background (n)%		
Australian	(11) 73%	(10) 66.6%
Other	(4) 26.6%	(5) 33.3%
Ambulation status (n)%		
Able to walk independently without aid	(15) 100%	(15) 100%
MS type (n) %		
Relapse remitting	(15) 100%	(15) 100%
Currently taking MS disease modifying medication		
Yes (n)%	(12) 80%	(10) 66.7%
Currently taking antidepressant medication		
Yes (n)%	(6) 40%	(5) 33.3%
How long on this medication in months (M, SD)	24.33 (20.68)	25.20 (15.53)
Previously diagnosed with depression		
Yes (n)%	(8) 53.3%	(7) 46.7%
Previously diagnosed with anxiety		
Yes (n)%	(6) 40%	(6) 40%

*M* Mean, *SD* standard deviation, *TAU* Treatment as usual control group

### Analysis of primary outcomes

Results of ANCOVAs examining group differences on primary and secondary variables at pre, post and 20 week follow up are presented in Table 2. There were no significant differences across the groups on demographic characteristics or significant differences on baseline levels of the primary and secondary outcome measures. Linear models revealed that those in the CBT group, when compared to the TAU group, had significantly lower scores on the BDI-II at post and at 20 week follow up (*d*s ranging from 1.34 to 1.66).

**Table 2:** Results of ANCOVAs examining group differences on primary and secondary variables at pre, post and 20 week follow up

	Pre	Post		Pre to post	20 week fup		Pre to fup
Variable	*Mean (SD)*	*Mean (SD)*	*F (df = 1,29)*	*d (95% CI)*	*Mean (SD)*	*F (df = 1,29)*	*d (95% CI)*
BDI-2							
CBT	29.80 (11.40)	10.89 (6.19)			9.33 (7.89)		
TAU	28.53 (12.21)	25.40 (10.31)	29.17***	1.66 (0.83, 2.49)	24.34 (13.54)	18.43***	1.34 (0.54, 2.13)
STAI							
CBT	45.40 (6.26)	33.87 (6.88)			36.20 (10.77)		
TAU	48.86 (11.20)	50.46 (12.22)	28.67***	1.63 (0.80, 2.45)	45.67 (14.15)	2.95	0.73 (− 0.01, 1.47)
MFIS							
CBT	12.13 (3.58)	8.73 (3.58)			8.06 (3.03)		
TAU	12.26 (3.84)	11.93 (4.38)	5.89*	0.78 (0.04, 1.52)	11.06 (4.74)	11.16**	0.73 (− 0.01, 1.47)
PES							
CBT	17.06 (5.62)	12.53 (6.18)			11.26 (4.51)		
TAU	18.80 (6.57)	17.67 (7.05)	3.92*	0.75 (0.01, 1.49)	16.80 (5.91)	9.80**	1.03 (0.26, 1.79)
MSQOL mental							
CBT	36.06 (14.81)	63.13 (14.42)			69.93 (19.64)		
TAU	40.06 (17.35)	44.20 (21.05)	12.73***	1.03 (0.26, 1.79)	49.27 (20.49)	12.28**	1.00 (0.24,1.76)
MSQOL physical							
CBT	47.39 (18.07)	65.92 (14.66)			63.32 (17.25)		
TAU	43.28 (17.63)	47.74 (19.07)	18.26***	1.04 (0.28, 1.80)	49.33 (21.32)	7.85**	0.70 (−0.03, 1.44)
PSQI							
CBT	9.00 (3.46)	4.40 (2.09)			4.80 (2.54)		
TAU	9.06 (3.93)	7.87 (2.99)	22.02***	1.31 (0.52, 2.10)	8.20 (3.60)	11.06**	1.06 (0.30,1.83)
PSSS							
CBT	63.73 (16.41)	59.46 (13.45)			62.26 (20.39)		
TAU	69.40 (12.10)	57.33 (12.48)	15.51**	0.96 (0.20, 1.71)	58.86 (17.31)	0.001	0.17 (−0.54, 0.89)
RSA							
CBT	137.29 (34.38)	165.45 (26.30)			164.10 (25.95)		
TAU	146.40 (32.38)	148.25 (32.63)	17.98***	0.57 (−0.16, 1.30)	149.53 (37.84)	8.49**	0.44 (−0.29, 1.16)
Acceptance							
CBT	30.40 (6.04)	28.20 (3.89)			27.93 (3.88)		
TAU	32.13 (4.82)	31.14 (4.78)	2.18	0.66 (−0.09, 1.41)	31.40 (5.96)	3.01	−0.67 (−1.39,0.08)
Problem solving							
CBT	6.76 (3.19)	8.87 (3.54)			8.93 (3.53)		
TAU	6.73 (4.10)	8.73 (4.31)	0.09	−0.46 (−1.19,0.26)	6.81 (3.97)	3.99*	0.55 (−0.18,1.28)
Avoidance							
CBT	9.87 (4.99)	4.20 (3.07)			3.93 (2.96)		
TAU	8.53 (5.22)	7.40 (4.45)	11.27**	0.81 (0.05, 1.53)	7.87 (5.24)	9.75**	0.90 (0.13, 1.62)

*FU* 20 week follow up, *ns* not significant, *CBT* tailored CBT intervention, *TAU* treatment as usual, *BDI* Beck Depression Inventory –II, *STAI* State Trait Anxiety Inventory, *MFIS* Modified Fatigue Impact Scale, *PES* Pain Effects Scale, *MSQOL mental* Multiple Sclerosis Quality of Life-mental health summary score, *MSQOL physical* Multiple Sclerosis Quality of Life-physical health summary score, *PSQI* Pittsburgh Sleep Quality Index, *PSSS* Perceived Social Support Scale, *RSA* Resilience Scale for Adults, *Acceptance* Acceptance of Chronic Health Conditions Scale, *Problem solving* Ways of coping questionnaire – planful problem-solving subscale, *Avoidance* Ways of coping questionnaire – escape-avoidance subscale

*p < .05, **p < .01; ***p < .001

### Analysis of secondary outcomes

Linear models revealed that those in the CBT group were associated with lower post intervention and 20 week follow up STAI scores (*d*s ranging from 0.73 to 1.63) although this was not statistically significant (*F*(1, 29) = 2.95, *p* > 0.05) compared to the TAU group. There were significant group differences on the MFIS and PES scores at post (*d*s ranging from 0.75 to 0.78) and 20 week follow up (*d*s ranging from 0.73 to 1.03) with the CBT group showing significantly greater reductions on scores on both scales.

There were significant group differences on the MSQOL physical health composite score at post and at 20 week follow up (*d*s ranging from 0.70 to 1.04) and the MSQOL mental health composite score at post and at 20 week follow up (*d*s ranging from 1.00 to 1.03) with the CBT group showing significantly greater improvements on both scales. There was also a significant group difference on the RSA at post and at 20 week follow up (*d*s ranging from 0.44 to 0.57). Specifically, there were significant group differences on the RSA subscale of personal competence at post (*F*(1, 29) = 10.54, *p* < 0.001) and 20 week follow up (*F*(1, 29) = 8.20, *p* < 0.01) and on the RSA subscale of social resources at post (*F*(1, 29) = 17.97, *p* < 0.001) and at 20 week follow up (*F*(1, 29) = 7.70, *p* < 0.01).

There were significant group differences on the PSQI score at post and at 20 week follow up (*d*s ranging from 1.06 to 1.31). There were also significant group differences on the PSSS score at post and at 20 week follow up (*d*s ranging from 0.17 to 0.96). Compared to the TAU group, those in the intervention showed more MS diagnosis acceptance although this was not statistically significant. Those in the CBT group also reported significantly lower escape avoidance coping at post and at 20 week follow up and significantly higher planful problem solving coping at 20 week follow up (*d*s ranging from −0.46 to 0.90). No other group differences were found for the other coping styles.

### Acceptance and satisfaction with the intervention

All participants in the intervention (*n* = 15) reported having a very strong therapeutic alliance with the psychologist (X = 102.33, SD = 9.59). All participants in the CBT intervention reported reading and completing all of the materials provided for homework and reported that the intervention was *‘very useful’*, that all of the 8 intervention sessions were ‘*much to very much’* useful and beneficial, that they would *‘definitely’* recommend the intervention to a close friend who had MS and that the early intervention should be offered as part of routine care straight after diagnosis. The majority of participants (12/15, 80%) reported that the intervention contained ‘*enough sessions*’, that they would like ‘*more sessions if they experienced new problems*’, that the intervention addressed their problems ‘*completely*’ (11/15, 73.4%) and that the sessions were ‘*completely- to a large extent*’ (11/15, 73.4%) as they expected them to be. Just over half of the participants reported that the intervention came ‘*at the right time*’ for them (8/15, 53.3%) with the other half of participants reporting that it ‘*could have even been offered earlier*’ (7/15, 46.7%).

## Discussion

This study demonstrated that a tailored 8-week individual face-to-face CBT based intervention was effective in significantly reducing depressive symptoms in patients newly diagnosed with MS. The data suggests that those participants in the CBT intervention reported significant reductions in levels of depressive symptoms. The between group effect sizes for the BDI-II at the end of treatment was 1.66 and 1.34 at 20 weeks follow up which were well above the 0.80 cut-off for a large treatment effect [39]. In addition, those in the CBT group reported significant reductions in level of anxiety, fatigue and pain impact on physical, cognitive and psychosocial functioning and escape avoidance coping and significant increases in MS related quality of life in both the mental and physical domains, MS illness acceptance, sleep quality, level of resilience in particular the personal competence and social resources domains, perceived social support and planful problem solving coping at both post and at the 20 week follow up assessment. The between group effect sizes at post treatment (range between 0.57 and 1.63) and at 20 weeks follow up (range between 0.44 and 1.06) strongly supported the benefits of early intervention.

Individuals in the CBT intervention reported being satisfied with the tailored 8-week intervention and adhered to the therapy. All participants found the intervention ‘*acceptable*’ and ‘*very useful*’. All participants reported that the intervention was timely, that it addressed their problems ‘*completely*’, met their expectations ‘*completely and to a large extent*’, that all materials and strategies based on CBT principles contained in all sessions were ‘*much-very much*’ useful and beneficial. Of note, participants indicated that the CBT intervention should be offered as part of routine care to everyone who is newly diagnosed with MS. Adherence data suggested that all participants in the CBT intervention completed all of the 8 modules including all the readings and homework and there were no drop outs suggesting individuals were self-motivated to receive the treatment. All participants also reported a strong therapeutic alliance with their therapist.

### Strengths and limitations

This research has several strengths. First, this study used a randomized, controlled pilot intervention design with clearly defined outcomes, and to the best of our knowledge, the first study to test an early tailored CBT intervention for the treatment of depressive symptoms in individuals who are within five years of being diagnosed with MS. Second, the research was conducted within a large MS clinic in a hospital service increasing the likelihood that the findings may be applicable to a wider MS population. Third, the sample was homogenous in terms of age, level of ambulation, length of MS diagnosis, MS type, education level and marital status. There were some limitations to this research. As a preliminary pilot study, it had a small sample size. Only two therapists conducted the CBT intervention. The majority of the study sample was educated and employed which may limit the generalizability of the results. The study mostly relied on self-report measures and as such there is a chance that participants may under or over report their symptoms. The current study did not compare the CBT intervention to an active comparative intervention. Future research should consider comparing the current treatment to a supportive counselling intervention which may control for non-specific therapeutic effects such as time and attention from a caring health professional. This will help to determine whether the specific components included in the CBT intervention are more effective in treating depression and resulting in broader based improvements or whether the therapeutic elements of seeing a psychologist, participating in a research study or coming in for regular appointments are just as effective in producing these improvements in those newly diagnosed with MS. The assessment period was limited to 3 months and it is not known if the benefits are sustained or if further ‘top up’ therapy is required. Larger studies with extended follow up and adequate disease measures are required in order to determine if the current intervention can impact disease progression. Monitoring of treatment fidelity (treatment implementation) was not done by an independent rater and no audio recordings of therapy sessions were undertaken which did not allow for therapist competency ratings. Also there were only two clinical psychologists who undertook all assessments and provided the intervention. In addition, future trials should include blinding of psychologists undertaking assessments and those providing the intervention and have separate people involved with the generation and allocation concealment, enrollment of participants and implementation and monitoring of the intervention. Further studies are required to assess the cost effectiveness and efficacy of the tailored CBT intervention among larger samples in order to promote it as part of routine care for individuals newly diagnosed with MS.

## Conclusion

The current preliminary results provide novel evidence for the benefits of early intervention for the treatment of depressive symptoms in patients newly diagnosed with MS. The tailored early CBT intervention was found to be an acceptable and effective treatment for depression in a sample of individuals newly diagnosed with MS within a hospital outpatient facility. It also had broader benefits on anxiety, fatigue and pain management, MS illness acceptance, sleep quality, MS related quality of life, coping, resilience and social support in these individuals.

## Abbreviations

ACHC: Acceptance of chronic health conditions scale
ANCOVA: Analysis of covariance
ANOVA: Analysis of variance
BDI-II: Beck depression inventory – version 2
CBT: Cognitive behavioural therapy
EDSS: Expanded disability status scale
HAQ-II: Helping alliance questionnaire -version 2
MFIS – 5: Modified fatigue impact scale – 5 item
MS: Multiple sclerosis
MSQOL-54: Multiple sclerosis quality of life – 54 item
PES: Pain effects scale
PSQI: Pittsburgh sleep quality index
PSSS: Perceived social support scale
RCT: Randomized controlled trial
RSA: Resilience scale for adults
SCID/IP: Structural clinical interview of disorders – research version
SPSS: Statistical package for social sciences
STAI: State trait anxiety inventory
TAU: Treatment as usual
WCQ: Ways of coping questionnaire

## References

[1] Australian Bureau of Statistics: *Disability, Ageing and Carers, Australia: Summary of Findings*. Catalogue No: 4430.0. Canberra: Australia; 2012.

[2] Giordano A, Granella F, Lugaresi A, Martinelli V, Trojano M, Confalonieri P, Radice D, Solari A, SIMS-Trial group (2011). Anxiety and depression in multiple sclerosis patients around diagnosis. J Neurol Sci.

[3] Janssens ACJW, Buljevac D, van Doorn PA, van der Meche FGA, Polman CH, Passchier J (2006). Prediction of anxiety and distress following diagnosis of multiple sclerosis: a two-year longitudinal study. Mult Scler.

[4] Tan-Kristanto S, Kiropoulos LA (2015). Resilience, self-efficacy, coping styles and depressive and anxiety symptoms in those newly diagnosed with multiple sclerosis. Psychol Health Med.

[5] Sadovnick AD, Eisen K, Ebers GC, Paty DW (1991). Cause of death in patients attending multiple sclerosis clinics. Neurology.

[6] Siegert RJ, Abernathy DA (2005). Depression in multiple sclerosis: a review. J Neurol Neurosurg Psychiatry.

[7] Dahl OP, Stordal E, Lydersen S, Midgard R (2009). Anxiety and depression in multiple sclerosis. A comparative population-based study in Nord-Troondelag County, Norway. Mult Scler.

[8] Gay MC, Vrignaud P, Garitte C, Meunier C (2010). Predictors of depression in multiple sclerosis patients. Acta Neurol Scand.

[9] Wood B, van der Mei IAF, Ponsonby AL, Pittas F, Quinn S, Dwyer T (2012). Prevalence and concurrence of anxiety, depression, and fatigue over time in multiple sclerosis. Mult Scler.

[10] Burns MN, Siddique J, Fokuo JK, Mohr DC (2010). Comorbid anxiety disorders and treatment of depression in people with multiple sclerosis. Rehabil Psychol.

[11] Janssens A, van Dorn P, de Boer J, van der Meche F, Passchier J, Hintzen R (2003). Impact of recently diagnosed multiple sclerosis on quality of life, anxiety, depression and distress of patients and partners. Acta Neurol Scand.

[12] Hind D, Cotter J, Thake A, Bradburn M, Cooper C, Isaac C, House A (2014). Cognitive behavioural therapy for the treatment of depression in people with multiple sclerosis: a systematic review and meta-analysis. BMC Psychiatry.

[13] Mohr DC, Boudewyn AC, Goodkin DE, Bostrom A, Epstein L (2001). Comparative outcomes for cognitive-behaviour therapy, supportive-expressive group psychotherapy and sertraline for the treatment of depression in multiple sclerosis. J Consult Clin Psychol.

[14] Askey-Jones S, David AS, Silber E, Shaw P, Chalder T (2013). Cognitive behavior therapy for common mental disorders in people with multiple sclerosis: a bench marking study. Behav Res Ther.

[15] Fischer A, Schroder J, Vettorazzi E, Wolf OT, Pottgen J, Lau S, Heesen C, Moritz S, Gold SM (2015). An online programme to reduce depression in patients with multiple sclerosis: a randomized controlled trial. Lancet Psychiatry.

[16] Lode K, Bru E, Klevan G, Myhr KM, Nyland H, Larsen JP (2009). Depressive symptoms and coping in newly diagnosed patients with multiple sclerosis. Mult Scler.

[17] Bronnum-Hansen H, Stenager E, Nylev Stenager E, Koch-Henriksen N (2005). Suicide among Danes with multiple sclerosis. J Neurol Neurosurg Psychiatry.

[18] Mohr DC, Goodkin DE (1999). Treatment of depression in multiple sclerosis, review and meta-analysis. Clin Psychol Sci and Pract.

[19] Lu CZ, Jensen MA, Arnason BG (1993). Interferon gamma- and interleukin-4 secreting cells in multiple sclerosis. J Neuroimmunol.

[20] Pujol J, Bello J, Deus J, Marti-Vilalta JL, Capdevilla A (1997). Lesions in the left arcuate fasciculus region and depressive symptoms in multiple sclerosis. Neurology.

[21] Mohr DC, Goodkin DE, Likosky W, Gatto N, Neilley LK, Griffen C, Stiebling B (1996). Therapeutic expectations of patients with multiple sclerosis upon initiating interferon beta-1b: Relationships to adherence to treatment. Mult Scler.

[22] Kern S, Ziemssen T (2008). Review: brain immune communication psychoneuroimmunology of multiple sclerosis. Mult Scler.

[23] Mohr DC, Hart SL, Goldberg A (2003). Effects of treatment for depression on fatigue in multiple sclerosis. Psychos Med.

[24] Mohr DC, Lover J, Brown T (2012). A randomized trial of stress management for the prevention of new brain lesions in MS. Neurology.

[25] Rost K, Smith JL, Dickinson M (2004). The effect of improving primary care depression management on employee absenteeism and productivity. A randomized trial. Med Care.

[26] Beck AT, Steer RA, Brown GK (1996). Beck depression inventory-second edition manual.

[27] Lancaster GA, Dodd S, Williamson PR (2004). Design and analysis of pilot studies: recommendations for good practice. J Eval Clin Pract.

[28] First MB, Spitzer RL, Gibbon M, Williams JBW (2002). Structured clinical interview for DSM-IV-TR axis I disorders, research version, patient edition. (SCID-I/P).

[29] Bowen J, Gibbons L, Gianas A, Kraft GH (2001). Self-administered expanded disability status scale with functional system scores correlates well with a physician-administered test. Mult Scler.

[30] Spielberger CD, Gorsuch RL, Lushene RE (1970). STAI manual for the state-trait anxiety inventory.

[31] Ritvo PG, Fischer JS, Miller DM, Andrews H, Paty DW, LaRocca NG (1997). Multiple sclerosis quality of life inventory: a user’s manual.

[32] Vickrey BG, Hays RD, Harooni R, Myers LW, Ellison GW (1995). A health-related quality of life measure for multiple sclerosis. Qual Life Res.

[33] Buysse DJ, Reynolds CF, Monk TH, Berman SR, Kupfer DJ (1989). The Pittsburgh sleep quality index: a new instrument for psychiatric practice and research. Psychiatry Res.

[34] Folkman S, Lazarus R. *Ways of Coping Questionnaire: Manual.* Mind Garden, Inc. Consulting Psychologists Press; 1988.

[35] Stuifbergen A, Becker H, Blozis S, Beal C, Park I (2008). Conceptualization and development of the acceptance of chronic health conditions scale. Issues Ment Health Nurs.

[36] Blumenthal JA, Burg MM, Barefoot J, Williams RB, Haney T, Zimet G (1997). Social support, type A behavior and coronary heart disease. Psychosom Med.

[37] Friborg O, Barlaug D, Martinussen M, Rosenvinge JH, Hjemdal O (2005). Resilience in relation to personality and intelligence. Int J Method Psychiatr Res.

[38] Luborsky L, Barber JP, Siqueland L, Johnson S, Najavits LM, Frank A, Daley D (1996). The revised helping alliance questionnaire (HAQ-II). J Psychother Pract Res.

[39] Cohen J (1988). Statistical power for the behavioural sciences.

